# Characteristics of site-specific response using the measured data from seismic accelerometers in Pohang Yeongil New Port under 9.12 and Pohang earthquakes

**DOI:** 10.1038/s41598-022-21862-5

**Published:** 2022-11-10

**Authors:** Jihye Seo, Deokhee Won

**Affiliations:** 1 Research Scientist at Coastal & Ocean Engineering Division, Korea Institute of Ocean Science & Technology (KIOST), Busan, Republic of Korea; 2 Department of Civil Engineering, Halla University, Wonju, Republic of Korea

**Keywords:** Natural hazards, Engineering

## Abstract

Recently, medium-sized earthquakes such as the Gyeongju 9.12 earthquake (September 12, 2016, M_L_ = 5.8) and the Pohang earthquake (November 15, 2017, M_L_ = 5.4) occurred in Korea, thereby increasing social concern about earthquakes. Because Korea is not located near the Circum-Pacific Belt, also referred to as the “Ring of Fire”, people in Korea are not used to earthquake disasters. Coastal areas in Korea are lined with multiple mega-cities and major industrial facilities. Most of them constructed on landfill, they have a high threat level to the damage to populations and structures leading to complex disasters in the event of an earthquake. However, studies of seismic hazards in ports is incomplete compared with those of onshore sites. Improving the understanding of seismic hazards and characterizing them catching up with related research from various perspectives being actively conducted. In this study, the site-specific response characteristics of the Pohang International Container Terminal in Pohang Yeongil New Port were analyzed using the S-wave energy of 10 sets of ground motions recorded by seismic accelerometers operated by the Ministry of Oceans and Fisheries based on the horizontal-to-vertical spectral ratio (H/V ratio) method. As a result of analysis, the H/V ratio curves show that the peak frequency values for the target site averages 9 Hz, and the natural period values of the site were preliminarily predicted to average 0.11 s. In addition, site amplification characteristics are different based on the seismic wave calculation method. The result can be used as data for identifying ground dynamic characteristics and verifying the site amplification coefficients and design spectrum in the seismic design.

## Introduction

For instrumental earthquakes (EQs), the seismic data can be analyzed from data recorded by seismometers. In the case of the Republic of Korea, the Korea Meteorological Administration (KMA) started officially seismic measurement in 1978. In the 1990s, digital instrument observation began to be conducted mainly by the Korea Institute of Geoscience and Mineral Resources^1^. Since the beginning of these instrumental observations, the largest magnitude EQ was occurred on September 12, 2016 (9.12 EQ, M_L_ = 5.8), which raised awareness that the Korean Peninsula is no longer safe from EQs^2^. Then, when the Pohang EQ that occurred the following year (PH EQ, November 11, 2017, M_L_ = 5.4) caused enormous social and economic damage, it further raised public concern about the dangers of EQ disasters. The Korean Peninsula is in a situation where 99.7% of all imports and exports are transported by shipping^3^. Thus, national trade ports are representative infrastructures and are highly significant for securing the stability of national logistics and transportation against natural disasters. Ports are one of the most vulnerable coastal utilities and need to be protected against their devastating effects of EQs. Unlike other natural disasters that can be partially predicted and prepared for, such as typhoons, heavy rains, heavy snowfall, cold waves, and landslides, seismic disasters cannot realistically be predicted in time and space. Therefore, it is necessary to develop a method that can minimize the loss of lives and property damage through rapid response by applying observation technology through monitoring.

**Figure 1:**
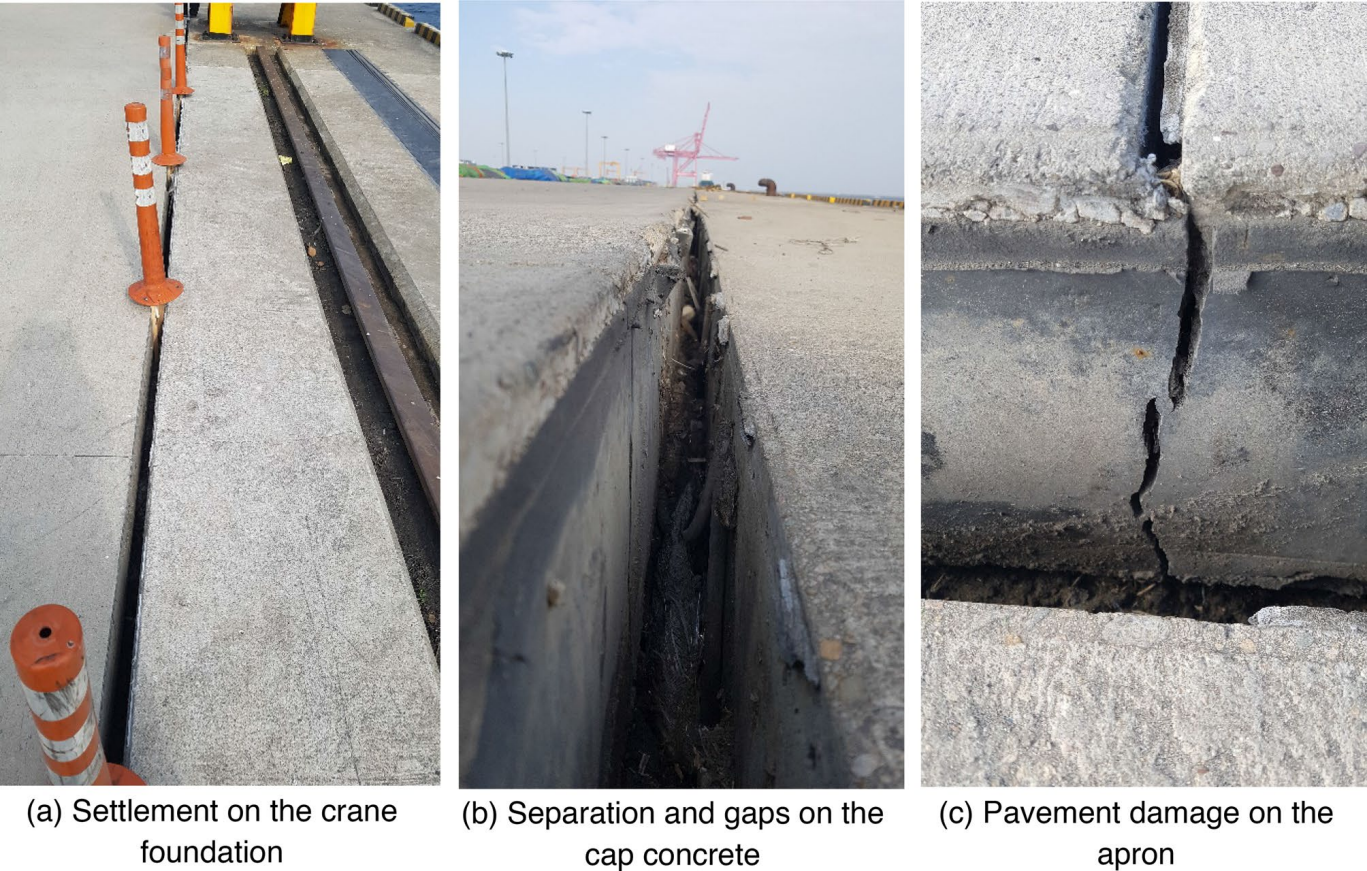
Earthquake damage in Pohang International Container Terminal.

To this end, the Ministry of Oceans and Fisheries (MOF) in Korea is operating the port alert system for seismic response by installing seismometers at national trade ports across the country. To establish an EQ database in ports, continuous efforts are being made to develop data processing techniques and to strengthen the information delivery service on seismic response in ports^4^. This system was used to assess and respond to emergency caused by the PH EQ in 2017. Significant damage occurred on the major facilities, which are the quay walls and cranes, of the Pohang International Container Terminal at Pohang Yeongil New Port (Fig. 1). The damage reportedly amounted to about $ 6.1 million, and a series of restoration works have been completed without secondary damage and are being operated normally. A port consists of extensive facilities that are vulnerable to EQs, but the damage to the quay structure has a catastrophic effect on the port management. In addition, cranes, levees, and utility (water, electricity and gas) lines are considered within the scope of EQ-induced damage cases for port facilities^5^. In line with the global trend of the “Fourth Industrial Revolution”, Korea has developed a road map to a smart port by 2030 based on the policy direction. Accordingly, research is being conducted to build a sustainable port by minimizing supply disruptions for digitalized ports and securing continuity of logistics functions on EQ disasters^6^.

In the soft ground, depending on the site-effect, even an EQ of the same magnitude can be amplified at a specific frequency, leading to a major disaster. The most famous instance is the Michoacan EQ with a moment magnitude of 8.0, which occurred in Mexico on September 19, 1985^7^. Differential settlement occurred at the foundation owing to the large amplification in the low-frequency band. This can also be found in San Francisco Bay, during the M_w_ = 6.9 Loma Prieta earthquake EQ of 17 October 1989^8^. Also, an extensive soil liquefaction was observed during the 1995 Kobe earthquake^9^. Lateral spreading in the storage yards at Port-au-Prince port occurred by sediment-induced amplification during the M_w_ 7.0 Haiti earthquake in 2010^10^. Such characteristics of ground amplification have been mainly studied in the field of natural sciences for seismological observation, which improves the accuracy of seismic sources by reducing local site-effects^11^. From the engineering perspective, it is also important to investigate ground motion attenuation and ground amplification effects according to the ground properties essential for seismic design.

There was a lack of real EQ records to be reflected in the domestic seismic design standards, particularly in Korea. For a more rational seismic design, the necessity of a design response spectrum suitable for domestic ground characteristics has been revealed in literature reviews^12–15^. Research related to seismic amplification or liquefaction on port area sites is still at an early stage. This is because the history of strong ground motion data in the Korean Peninsula is small owing to the low incidence of strong EQs, and it has been less than 10 years the digitized database of seismic measurement data in the port area began to be constructed. The first damage case in a port area since records began with instrumental observations in Korea was reported for the 2017 PH EQ. Thus, studies have been conducted to analyze the damage mechanism and the behavior of port structures^16,17^, and research on the earthquake-induced liquefaction is also being actively conducted^18^. In this study, based on the accelerograph record measured by the Port Alert System for Seismic Response operated by the MOF, the site response characteristics were analyzed around the port measuring station. A spectral ratio method was used, which is the horizontal component at the surface to the vertical component in the bedrock referred to as the “H/V ratio”. The response characteristics at Pohang Yeongil New Port, where the measuring station is located, were analyzed using 10 seismic motion data of the foreshock, mainshock and aftershocks during the 9.12 and PH EQs. The site-specific response for characteristics of seismic motion recorded at measuring stations of the surface and bedrock (borehole) was investigated.

## Analysis method

### Theoretical background

In research related to the site-effect using observed seismic waves, amplification characteristics are typically examined by the secondary wave (S-wave) and can be divided into the spectral, inverse calculation. and unknown term separation method^19^. Based on waveform types, in addition to the S-wave energy, background noises (microtremors) and the coda wave are used to analyze the spectrum. In particular, the horizontal-to-vertical spectral ratio (H/V ratio) method, which is widely used because of its speed and convenience, was employed to estimate the site effect evaluation^20,21^. A method that considers the H/V ratio of the target site to the reference site with well-developed outcrops as the amplification ratio of that site has been studied^22^. The method of using the H/V ratio by employing the three components observed at arbitrary sites^23,24^ has recently been applied to nuclear power plants, dams, reservoir, and major observatory sites in Korea^25–32^. In practice, it is often determined by the H/V ratio technique in which the peak frequency (${\varvec{f}}_{{\varvec{p}}}$) of recorded H/V ratio is interpreted as the resonance frequency. The H/V ratio is given as in Eq. (1) using the Fourier spectrum of the seismic motion.

1 $$\log \left( {{\varvec{H}}/{\varvec{V}}} \right)\user2{ } = \log \left( {\frac{{{\varvec{H}}_{{\varvec{E}}}^{2} + {\varvec{H}}_{{\varvec{N}}}^{2} }}{2}} \right) - \log \left( {{\varvec{V}}_{{\varvec{Z}}} } \right)\user2{ }$$

where, ${\varvec{H}}_{{\varvec{E}}}$ and ${\varvec{H}}_{{\varvec{N}}}$ represent the Fourier spectra of the horizontal components in the east–west and north–south directions of the measured seismic motion, and ${\varvec{V}}_{{\varvec{Z}}}$ is the Fourier spectrum of the vertical component of the seismic motion. In addition, *H* represents for the horizontal component of the target site and *V* represents the vertical component of the target site.

### Analysis materials

As of June 2021, the MOF in Korea has been operating the Port Alert System for Seismic Response in 28 stations in 13 ports, centered on trade ports across the country, such as Busan Port and Incheon Port. Among the other stations, the seismic motion data were collected from two measuring stations (PHF and PHT) installed in Pohang Port to understand the characteristics of the port site-specific response and to analyze the amplification quantitatively. A borehole seismometer is installed in the bedrock, which is less affected by the site amplification effect by the upper sedimentary layer or the background noise of the surface; therefore, it is possible to observe the seismic observations accurately compared with the surface-type seismometer. However, unlike in bedrock areas, in soils with a large site amplification, it is necessary to consider the site effect characteristics^33^. The aforementioned two seismic stations were separated by approximately 8.6 km in a straight line, and specific details are summarized in Table 1.

**Table 1:** List of seismic measuring stations.

Station	Name code	Location	Sensor type	Digitizer & acquisition	Altitude	Borehole depth
Reference	PHF	36.0458 N, 129.3767 E	CMG-5 TB	CMG-DM24S6EAM	11 m	48 m
Target	PHT	36.1093 N, 129.4345 E	CMG-5TC	CMG-DAS-S3	23 m	–

The seismic accelerometer at PHF as a reference station in this study is installed in the bedrock, and the ground layer formations are as follows. The ground layer consists of a reclamation layer with fine sand up to 1.5 m below the surface; from 1.5 to 38 m, it is composed of a marine sedimentary layer with silty clay, containing small amounts of fine gravel and shells in the lower part. The 38–42 m section is a soft rock layer composed of mudstone, and cracks and joints are developed. Finally, the 42–48 m section is composed of muddy hard rock, and a borehole accelerometer was installed nearly 48 m below^4^. In addition, the surface-type accelerometer at PHT as the target station for site amplification analysis is installed in the basement of the machine room in Pohang Yeongil New Port.

Table 2 summarizes the seismic motion data, including 20 horizontal and 10 vertical components for each measuring station. The reference station, PHF is approximately 36 and 7 km from the epicenter of the 9.12 and PH EQs, respectively. Moreover, the seismic motion used in the analysis has a signal-to-noise ratio of up to approximately 10,000 times and a minimum of approximately 1000 times or more. The amplification characteristics of the port site were analyzed based on these seismic motion data. As a result of the fault plane solution analysis, the 9.12 EQ showed typical the strike slip fault motion. However, in the PH EQ, the fault plane of the mainshock (PH EQ#1) was analyzed as predominantly right-lateral strike-slip and thrust movements in a northeast direction. Then, the aftershock with a magnitude of 4.3 (PH EQ#3) was analyzed as a reverse slip in the north-northeast direction. In other major aftershocks with a magnitude of 3.0 or greater, the strike slip component was dominant^34^.

**Table 2:** List of 9.12 and PH EQ occurrence.

No	DATE (YYYY/MM/DD HH:MM:SS)	Magnitude (ML)	Depth (km)	Location	Target station distance from epicenter (km)
9.12 EQ#1	2016/09/12 19:44:30	5.1	–	35.77 N, 129.19 E	43.67 km
9.12 EQ#2	2016/09/12 20:32:50	5.8	15	35.76 N, 129.19 E	44.64 km
9.12 EQ#3	2016/09/12 20:34:22	3.6	–	35.78 N, 129.19 E	42.72 km
9.12 EQ#4	2016/09/19 20:33:58	4.5	–	35.74 N, 129.18 E	47.04 km
9.12 EQ#5	2016/09/21 11:53:54	3.5	–	35.75 N, 129.18 E	46.05 km
PH EQ#1	2017/11/15 14:29:31	5.4	7	36.11 N, 129.37 E	5.79 km
PH EQ#2	2017/11/15 14:32:59	3.6	8	36.10 N, 129.36 E	6.76 km
PH EQ#3	2017/11/15 16:49:30	4.3	10	36.12 N, 129.36 E	6.80 km
PH EQ#4	2017/11/16 09:02:42	3.6	8	36.12 N, 129.37 E	5.91 km
PH EQ#5	2018/02/11 05:03:03	4.6	14	36.08 N, 129.33 E	9.95 km

### Procedure and verification of analysis

The seismic motion data used in this study were stored in the format of mini-SEED (Standard for the Exchange of Earthquake Data) obtained from the three-axis accelerometer, and the physical factor (pf) was applied to convert the acceleration values. The Nyquist frequency was set to 50 Hz because 100 samples per second (0.01 s) were sampled. Approximately 15 s to less than 20 s of the S-wave energy window were selected for analysis. Table 3 shows the procedure for obtaining the natural frequency of a site in the port area using the H/V ratio technique. After converting the data stored in mini-SEED format from the Port Alert System for Seismic Response of MOF to ASCII format, fast Fourier transform (FFT) was performed to obtain the spectrum data converted into the frequency domain, and was calculated the H/V ratio using Eq. (1). This is a procedure to determine a clear peak point as a natural frequency of the site by smoothing of the obtained H/V ratio on the seismic motion.

**Table 3:**
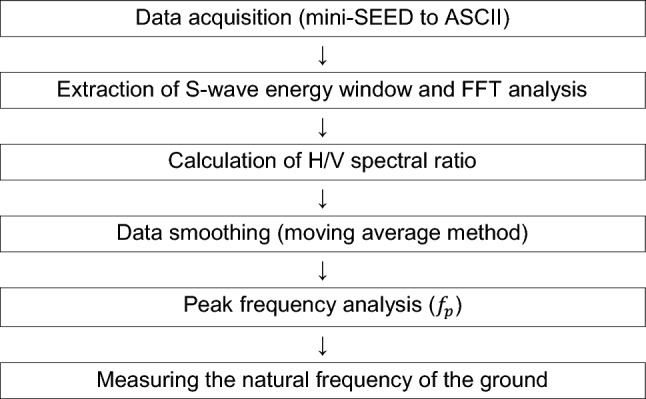
Calculation procedure of H/V Fourier spectral ratio.

**Figure 2:**
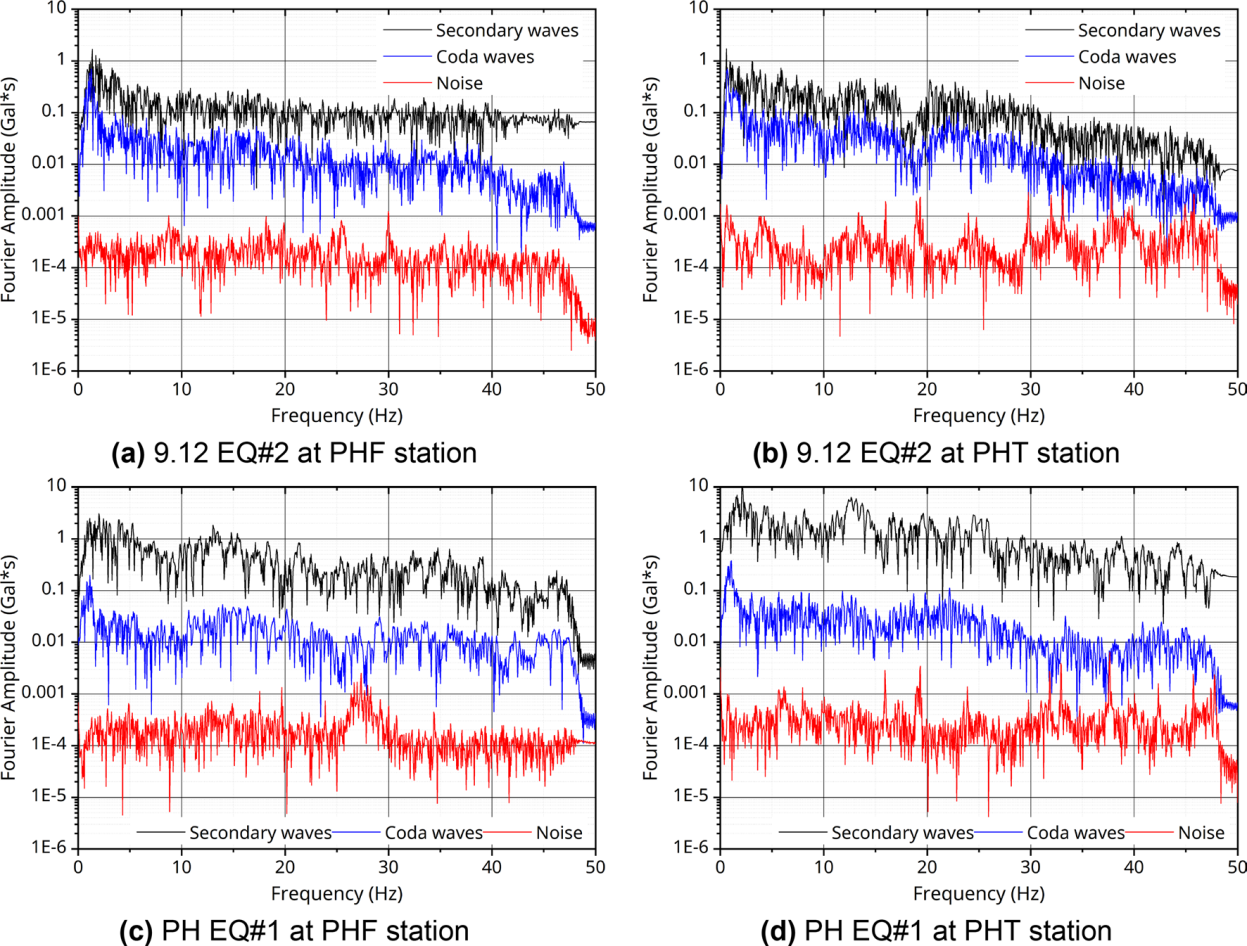
FFT analysis for Z-component of stations according to earthquakes (left: PHF, right: PHT).

Prior to the site-specific response analysis, the consistency of the energy magnitude according to the frequencies of acceleration waveforms was verified by analyzing from S-wave energy, coda waves, and background noise on the seismic motion data for each measurement station^29^. Ninety sets of data for each component and waveform type were verified from 10 EQ records. Here, the S-wave energy window was selected for approximately 15–20 s from the arrival time of the S-wave, and the coda wave window was sampled for 15–20 s after the point when the S-wave travel time was approximately double. In addition, the background noise was used for 15–20 s before the arrival of the P-wave. At the two stations (PHF and PHT) as shown in Fig. 2, the S-wave was the largest, followed by the coda wave and then background noise consistently. There was a slight difference depending on the measuring station and frequency range, but when the Fourier amplitude of the S-wave energy and the background noise were compared, the result was a difference of at least 1000 times. In particular, in the range of 10 Hz or less, which corresponds to the main frequency range of the site amplification effect, there was a large difference between the S-wave and the background noise from approximately 1000 to up to 10,000 times, but the trend for three waveform phase had mutual consistency. Therefore, the reliability of the analysis results on the H/V ratio was boosted based on these data.

## Analysis results

### Basic analysis of measurement records

**Figure 3:**
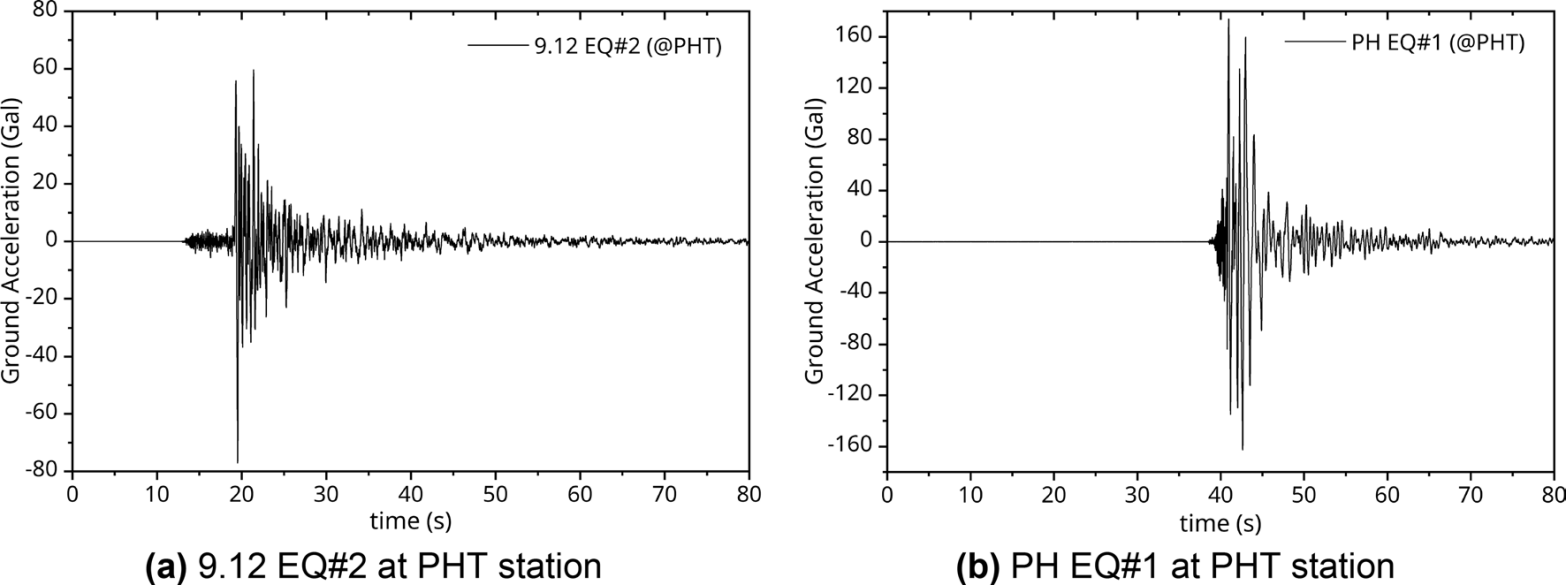
Wave time series for E-component at PHT according to the earthquake.

Seismic motion waves were measured in three directions: *E*, *N* and *Z* (x, y, and z-axis). Figures 3a and b show the wave time-series for the horizontal component of 9.12 EQ#2 and PH EQ#1 recorded at the PHT station, respectively. The peak ground acceleration level can be examined on seismic motion records for each EQ. The 9.12 EQ originated 15 km beneath the surface. It is a deeper than the average depth of EQs with a magnitude 5.0 or greater in Korea, which is 8.16 km. The strong motion duration was also measured for 1 to 2 s in the form of an impact wave in which energy was concentrated^34^. In the case of PH EQ#1, which was relatively close to the epicenter, the vector summation of three components measured at the PHF station reached maximum of 454.839 gal, as summarized in Table 4. In addition, the strong motion duration was quite short, as shown in Fig. 3b.

**Table 4:** Peak ground acceleration according to the station.

	PHF (gal = cm/s^2^)					PHT (gal = cm/s^2^)				
	E	N	Z	Hor	3COM	E	N	Z	Hor	3COM
9.12 EQ#1	− 15.309	13.180	− 7.441	20.201	25.242	− 44.152	− 32.300	− 16.017	54.705	57.002
9.12 EQ#2	− 39.161	− 34.931	20.430	52.477	66.267	− 76.998	− 60.925	21.776	98.186	100.572
9.12 EQ#3	− 1.294	− 1.294	0.882	1.831	2.409	− 2.656	− 2.495	− 1.083	3.644	3.802
9.12 EQ#4	− 8.713	7.521	4.825	11.510	14.572	− 23.051	− 18.345	8.224	29.460	30.586
9.12 EQ#5	− 1.451	1.061	0.744	1.798	2.216	− 1.910	− 1.441	− 0.817	2.393	2.528
PH EQ#1	− 324.776	− 305.848	− 88.633	446.119	454.839	173.960	233.614	217.828	291.269	363.712
PH EQ#2	− 17.455	− 18.975	− 6.802	25.782	26.664	9.892	− 11.463	− 9.055	15.141	17.642
PH EQ#3	− 40.164	23.785	− 18.558	46.678	50.232	− 34.843	40.804	77.364	53.656	94.150
PH EQ#4	− 13.026	− 13.538	− 6.745	18.787	19.961	25.312	− 29.509	40.395	38.878	56.065
PH EQ#5	119.416	− 72.490	− 46.491	139.696	147.229	35.948	− 51.827	41.477	63.074	75.489

**Figure 4:**
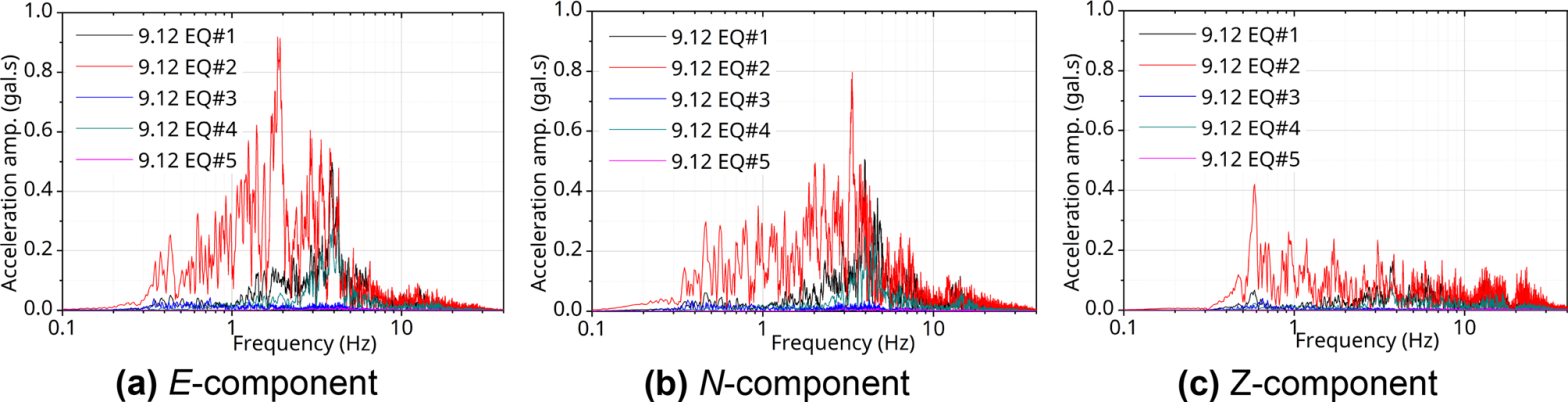
Frequency domain analysis @PHT with 9.12 EQs.

**Figure 5:**
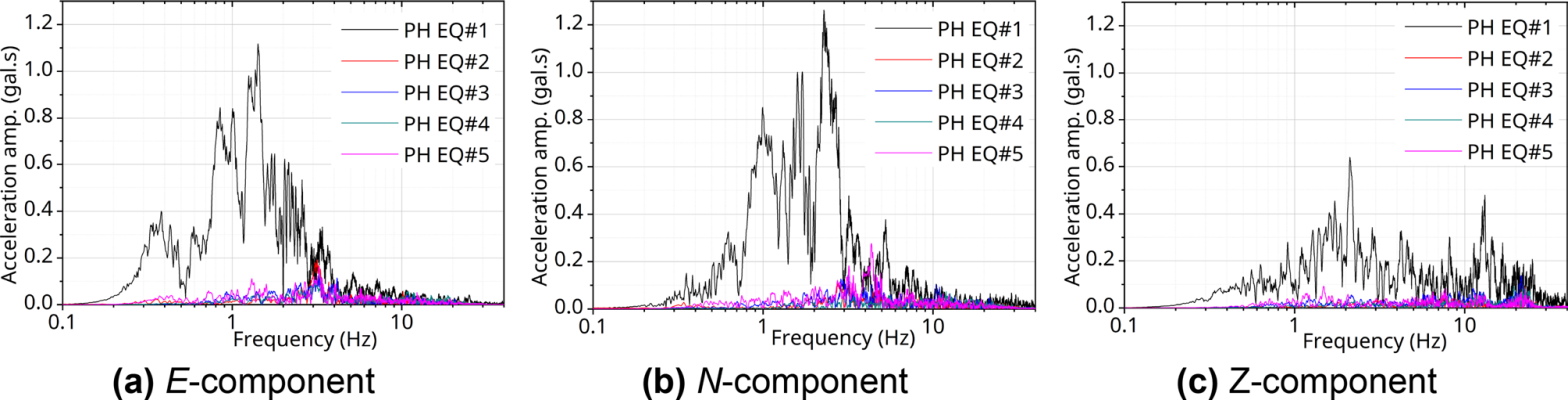
Frequency domain analysis @PHT with PH EQs.

Figures 4 and 5 show the seismic motion data in the frequency domain through Fourier spectrum analysis for all EQs used in the analysis at the target station, PHT. The vertical axis represents the Fourier amplitude on acceleration values, and the unit corresponds to [gal·s]. Figures 4a and 5a show the *E*-component in a horizontal direction for each seismic motion, Figs. 4b and 5b are the *N*-component in the horizontal direction, and Figs. 4c and 5c show are the *Z*-component in the vertical direction in the FFT result. The 9.12 EQ#2 had a large high-frequency energy, and, for the PH EQ#1, relatively low-frequency energy predominated. In particular, in the band below1.0 Hz, the amplitude of the horizontal components (*E*, *N*) of PH EQ#1 was approximately twice as large as that of the 9.12 EQ#2. In addition, for 9.12 EQ#2 and PH EQ#1, the peak frequency band of the *Z*-component was different from that of the *E*- and *N*-components. The high-frequency (short-period) component of the *Z*-component was predominant in the PH EQ#1 compared to the 9.12 EQ#2.

**Figure 6:**
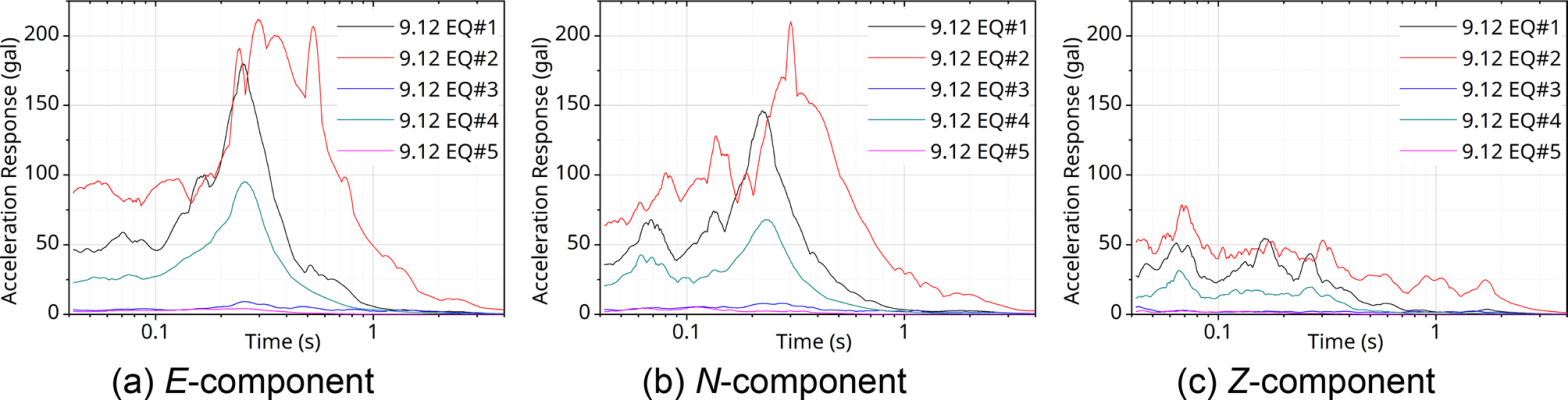
Response spectra @PHT with 9.12 EQs.

**Figure 7:**
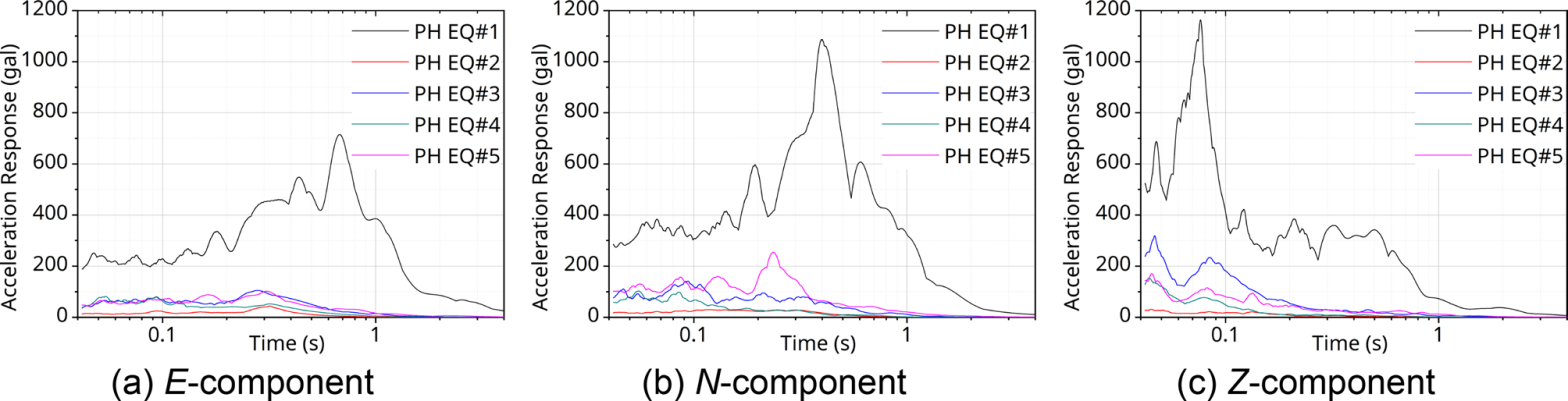
Response spectra @PHT with PH EQs.

**Figure 8:**
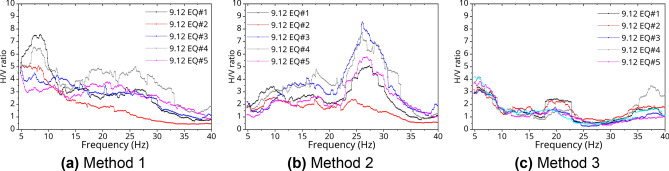
H/V Fourier spectra ratio with 9.12 EQs.

Figures 6 and 7 show the response spectra of the measured data from seismic accelerometers at the PHT station. Here, a damping factor was applied as 5%. Figures 6a, b, 7a, and b graph the result of horizontal components. Figures 7c and 8c indicate the vertical component. The result of the response spectrum of the 9.12 EQs is shown in Fig. 6. The period components were predominant in range of 0.07–0.7 s. In the case shown in Fig. 7 for the PH EQs, the peak periods were also diversely distributed as 0.06–0.7 s for each component (*E, N, Z*). In particular, for the vertical direction of the PH EQs, shown in Fig. 7c, the acceleration had larger amplitude than that of the 9.12 EQs, as shown in Fig. 6c. Using the analyzed results, it can evaluate the structural condition compared with the natural frequency characteristics of the structure applied to the seismic design. For reference, the natural frequency bands of the gravity-type caisson and the pier-type quay wall, which are representative port structures, are 1.29–1.63 Hz (0.61–0.78 s) and 0.66–3.73 Hz (0.27–1.52 s), respectively^35^.

### Site-specific response of Pohang Yeongil New Port based on seismic records

**Figure 9:**
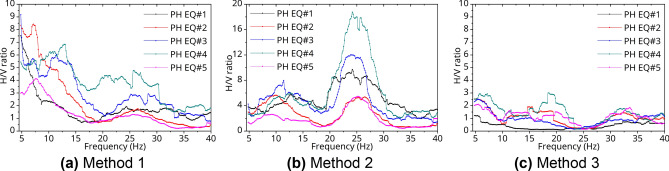
H/V Fourier spectra ratio with PH EQs.

The local site effect may cause amplification of seismic waves induced by EQs and plays an important role in site-specific ground motion predictions and seismic hazard assessments. To evaluate dynamic response characteristics considering structure-soil Interaction, seismic sources, and an attenuation in crusts, the site effect should be considered when deliberating how to do it. In this study, the three calculating methods for the site-specific response to determine the site effect were selected as follows^31^: (1) the first method (#1: ${\varvec{H}}_{{\varvec{t}}} /{\varvec{V}}_{{\varvec{r}}}$), using horizontal components of the PHT (the target station) to the vertical component of the PHF (the reference station), (2) the second method (#2: ${\varvec{V}}_{{\varvec{t}}} /{\varvec{V}}_{{\varvec{r}}}$), using only vertical components of the target station and the reference station, and (3) the third method (#3: $({\varvec{H}}_{{\varvec{t}}} /{\varvec{V}}_{{\varvec{t}}} )/({\varvec{H}}_{{\varvec{r}}} /{\varvec{V}}_{{\varvec{r}}}$), using the H/V ratio of the target station to the H/V ratio of the reference station. Figures 8 and 9 show the results of the H/V ratio at the Pohang International Container Terminal in Pohang Yeongil New Port for the S-wave phase of the 9.12 and the PH EQs when these methods were used. First, the amplification ratio of the site had a similar trend according to the all analyzed EQs and calculation methods. In addition, the epicenters of PH EQs were relatively close to the measuring stations as shown in Tables 2 and 3, so a larger H/V ratio could be obtained than that of 9.12 EQs because to the hypocentral parameter.

The peak frequency ($$f_{p} )$$ refers to the frequency band with a maximum value between 1 and 20 Hz in the H/V ratio graph, and it is also interpreted as the resonance frequency. In this study, the $$f_{p}$$ of the PHT is as follows. The $$f_{p}$$ obtained by Method 1 in Figs. 8a and 9a was in the range of 6–12 Hz (0.083–0.167 s), and the $$f_{p}$$ obtained by Method 2 ranged from 7 to 18 Hz (0.056–0.143 s), as shown in Figs. 8b and 9b. For Method 3 in Figs. 8c and 9c, it was in the range of 5–8 Hz (0.125–0.200 s). In the deep or soft soil, the peak frequency is generally observed in the lower frequency band. Therefore, the average peak frequency and natural period of the ground at PHT can be predicted as about 0.9 Hz and 0.11 s respectively. This is a result similar to that estimated the natural period for the inland areas of Korea as the 0.5 s range or less^14^.

There are two methods for time histories of a design ground motion to evaluate the seismic design and performance: a method using actual measurement records or an artificially synthesizing method compatible to the design response spectra. Recently, there has been a trend to use actual measurement records as the seismic acceleration time history to consider the impact on the site environment fully. However, in the case of Korean records, there are problems in that the intensity of the ground motion does not meet the design purpose or that the response spectra of the record are different form the shape of the design response spectra. Thus, to maintain the seismic wave characteristics and site characteristics and to examine appropriately the intensity and shape required for the seismic design and response spectra, it was compared with recorded data of the time history of the 9.12 EQs and the PH EQs. Here, the seismic performance objectives of structures in the target station were defined according to the domestic seismic design standards^36^ as a combination of a design ground motion with an average return period of 500 years of seismic Zone I and a seismic performance level requiring collapse prevention of a Grade II. According to the drilling results conducted by the Pohang Regional Office of Oceans and Fisheries^37^, in the site around PHT, the soil stratification was distributed in the order of a reclamation layer, sediment layer, weathering ground, and bedrock. The average shear wave velocity ($$V_{s} )$$, according to the suspension P-S logging test was, exceeded 260 m/s. Finally, the soil type can be classified with S2 as shallow and hard soil.

**Figure 10:**
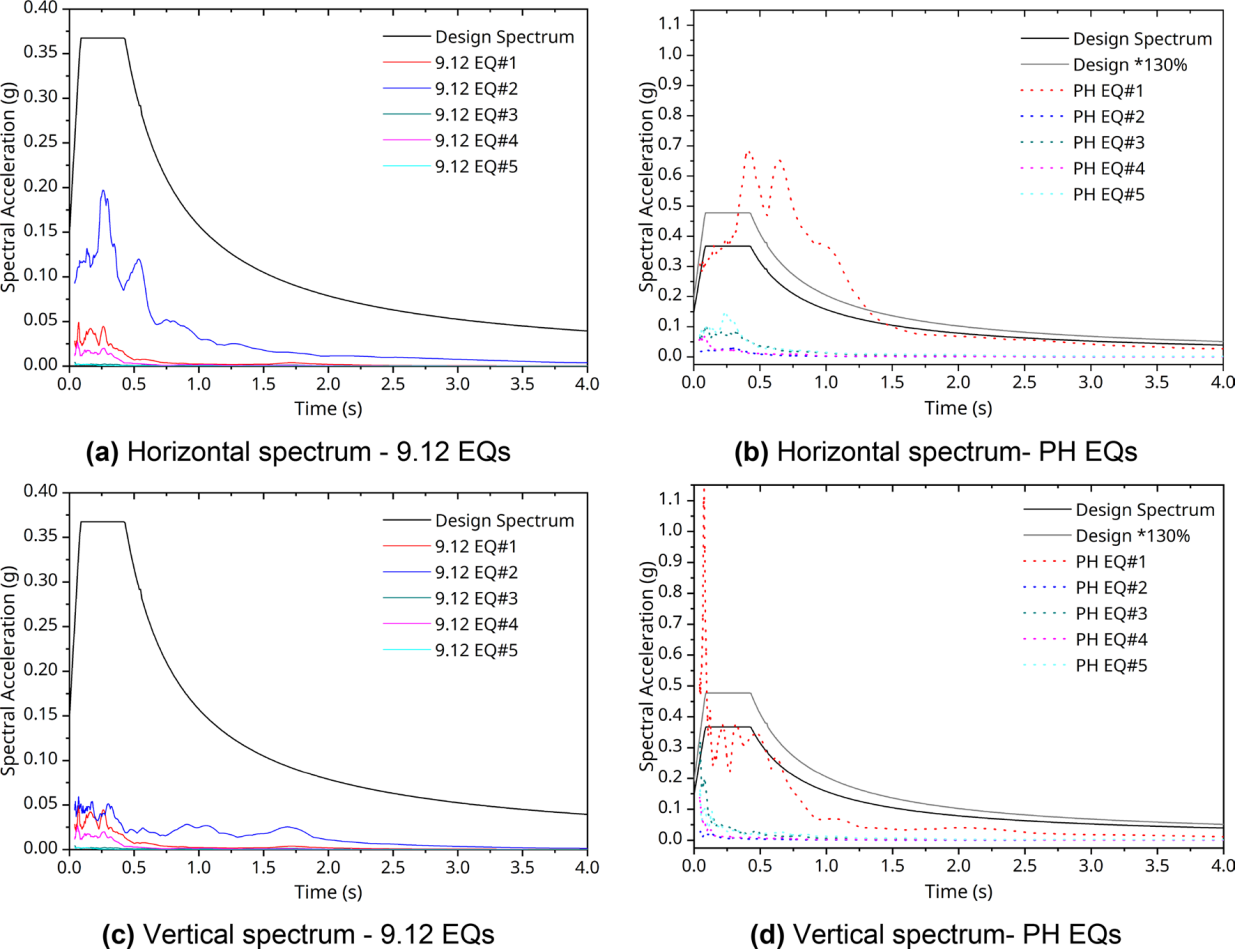
Comparison with the acceleration response spectra and design response spectra.

Based on this, the acceleration standard design response spectrum was obtained using the site coefficient according to the effective horizontal peak ground acceleration (0.105 g) on the bedrock of Pohang Yeongil New Port. In Fig. 10, the standard design response spectrum and the acceleration response spectra are compared using the measured data in Figs. 6 and 7. Here, the measurement data are the result of analyzing the acceleration response spectrum of the vertical and the horizontal components with 5% damping ratio. In the case of the 9.12 EQs, as shown in Fig. 10a and c, the horizontal and vertical spectra showed responses within the design response spectrum. However, in case of PH EQ#1, which was measured very close to the epicenter, the seismic motion had a level exceeding 1.3 times the design response spectrum of the site as shown in Fig. 10b and d. The response was clearly noticeable, ranging from 0.1 to 0.5 s, which can affect the port structure. Compared with the 9.12 EQs, the response to the vertical component was more pronounced in the very short period (high frequency) of 0.01 s, as shown in Fig. 10d. For the PH EQs identified as injection-induced EQs^38^, the vibration characteristics were different from those of the 9.12 EQs, which were natural EQs. As Fig. 3b shows, casualties were not heavy because of the extremely short strong motion duration. This can be confirmed in the H/V Fourier spectra ratio calculated by Method 2—see Fig. 9b—and the *Z*-component response spectra in Fig. 7c.

For the seismic wave from the El-centro Observatory on the 1940 Imperial Valley EQ, which is typically used for seismic design and response analysis, the strong motion duration was approximately 24.44 s, whereas the EQs used in this study had the form of shock waves with a short strong motion duration. It can be observed that the seismic wave characteristics recorded for ground motion are different and not compatible with the design response spectrum. Therefore, to generate the ground motion using these measuring records, it should be converted as a virtual ground motion appropriately according to synthesis conditions. In order to adequately model the standard deviation of the surface response spectra, at least 10 motions (and preferably 20) are required to obtain stable average spectral results^39^.

In the recent study of the response spectrum based on acceleration measurement data, it was found that the natural frequency, epicenter distance, and soil type of the site strongly influenced on the characteristics of the response spectrum, and the fault movement type of the seismic source, magnitude of the EQ, and depth of the sedimentary layer were less affected^40^. In addition, the engineering characteristics of vertical ground motion had emphasized in the seismic analysis and design^41^.

**Figure 11:**
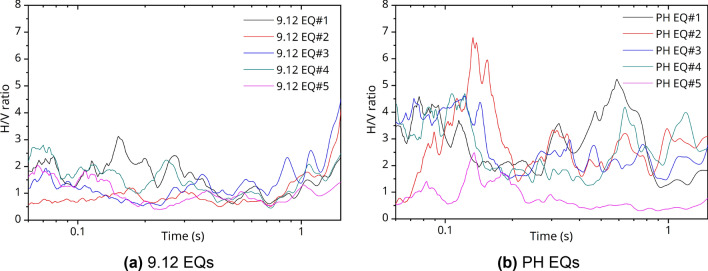
H/V response spectral ratio of Method 2 at PHT.

Figure 11 shows the H/V response spectral ratios of the site using only vertical components corresponding to Method 2, mentioned previously. Here, the H/V response spectral ratios were based on the method proposed in^42^ using an acceleration response spectrum with a damping ratio of 5%. The vertical axis represents the amplification ratio of the normalized response spectrum with respect to the maximum acceleration. The measured acceleration values of the seismic ground motion were calculated using geometric mean values. In the case of the 9.12 EQs, the response spectral ratio occurred up to three times in the short period range at approximately 0.1–0.3 s, and in the case of the PH EQs, it also occurred up to seven times in a short period of approximately 0.06–0.2 s. It was similar to the peak period derived from the H/V Fourier spectra ratio in Figs. 8b and 9b of 0.056–0.111 s.

For reference, background noise sources in the sub-1.0-s band include natural vibration sources, such as wind and waves, and artificial vibration sources, such as transportation and factories. In the period band of 1.0–10.0 s (0.1–1.0 Hz), the main source is a microseism such as the wave activity on the coast and tides^43^. Therefore, the measured data of the seismic accelerometers located in the port area may have been reflected in greater amplification.

## Summary and conclusion

In terms of an emergency response system and flexible decision-making support in the event of an EQ, the scientific and reasonable safety assessment technology underlying port areas, which is a representative infrastructure, is of major importance. This can cause significant secondary damage caused by downtime beyond damage to the facility itself. In Korea, the recent 9.12 and the PH EQs caused severe socio-economic damage, prompting concern about EQs. In countries near the Ring of Fire, such as the United States, Japan, and Chile, which have a high probability of EQ occurrence, infrastructure facilities were already managed based on probabilistic seismic hazard analysis and the ground motion prediction equation to prepare for and respond to EQ disasters. Research on the site effects has not been systematic in Korea, and it is necessary to study the local site-specific amplification to analyze seismic sources and an attenuation in crusts-related values to improve the assessment of seismic hazards and risks. In this study, the amplification response characteristics of the site in Pohang Yeongil New Port based on the H/V ratio technique were analyzed in an exemplary manner using the S-wave energy of the acceleration response signal obtained from the seismic accelerometer operated by the MOF, and it can be summarized as follows.

1. The response spectra on seismic waves showed that the high-frequency (short-period) component of the vertical direction was predominant in the PH EQ#1 compared to the 9.12 EQ#2.
2. As a result of analyzing the H/V Fourier spectra ratio of the PHT site, the peak frequency was observed from 6 to 12 Hz for Method 1, 7 to 18 Hz (0.056–0.143 s) for Method 2, and 5 to 8 Hz for Method 3.
3. As a result of analyzing the H/V response spectra ratio of the PHT site by Method 2, the amplitude occurred up to three times in the short period of approximately 0.1–0.3 s and up to seven times in the similar short period of approximately 0.06–0.2 s for the 9.12 EQs and PH EQs, respectively. This was similar to the peak period in the H/V Fourier spectra ratio analysis. However, the site amplification might have been reflected more greatly in the measured data of the seismic accelerometers located in the port because of the characteristics of the background noise with a specific period band.

This result of site responses can be used to build a database suitable for each geological condition and improve the regional and site coefficients. Furthermore, it can be used to estimate an advanced seismic hazard and develop a safety assessment technology utilizing of seismic accelerometers. After the outbreak of a large EQ, it is important to estimate strong ground motions at the site of damaged port structures to analyze the damage mechanism and determine the restoration policy. In particular, for a port composed of reclaimed ground, more reasonable and reliable characteristics of the port site can be obtained through the site amplification response analysis by also using measured data from a sand layer.

## Supplementary Information

Supplementary Information 1.

Supplementary Information 2.

Supplementary Information 3.

Supplementary Information 4.

Supplementary Information 5.

## Data Availability

All data generated or analysed during this study are included in this published article (and its supplementary information files).
