# Supplementary material for: Characteristics of site-specific response using the measured data from seismic accelerometers in Pohang Yeongil New Port under 9.12 and Pohang earthquakes
Source: Sci Rep. 2022 Nov 10;12:19249. doi: 10.1038/s41598-022-21862-5 (PMC9649761; doi:10.1038/s41598-022-21862-5)
Supplement: Supplementary file 5 — Supplementary Information 5. [file 41598_2022_21862_MOESM5_ESM.docx]

**Supplementary materials**

Characteristics of Site-Specific Response Using the Measured Data from Seismic Accelerometers in Pohang Yeongil New Port under 9.12 and Pohang Earthquakes

Jihye Seo^a^, Deok Hee Won^b^*

^a^ MS, Research Scientist at Coastal & Ocean Engineering Division, Korea Institute of Ocean Science & Technology (KIOST), Busan, Republic of Korea

^b^ Ph.D., Professor at Department of Civil Engineering, Halla University, Wonju, Republic of Korea

* thekey.won@halla.ac.kr

Supplemental Table 1: The Calibrated Parameters on Seismic Instruments

| Station Code | Sensor Type | Component | Sensor Sensitivity  (V/m/s^2^) | Digitizer & Acquisition Type | Digitizer & Acquisition Sensitivity  (μV/count) | Physical factor  (gal/count) |
| --- | --- | --- | --- | --- | --- | --- |
| PHF | CMG-5TB | Z | 2.038 | CMG-DM24S6EAM | 3.229 | 0.0001584 |
|  |  | N | 2.044 |  | 3.230 | 0.0001580 |
|  |  | E | 2.040 |  | 3.227 | 0.0001582 |
| PHT | CMG-5TC | Z | 1.022 | CMG-DAS-S3 | 3.218 | 0.0003149 |
|  |  | N | 1.018 |  | 3.221 | 0.0003164 |
|  |  | E | 1.020 |  | 3.221 | 0.0003158 |

Supplemental Figure 1: Time-averaged shear wave velocity (Vs) for profile


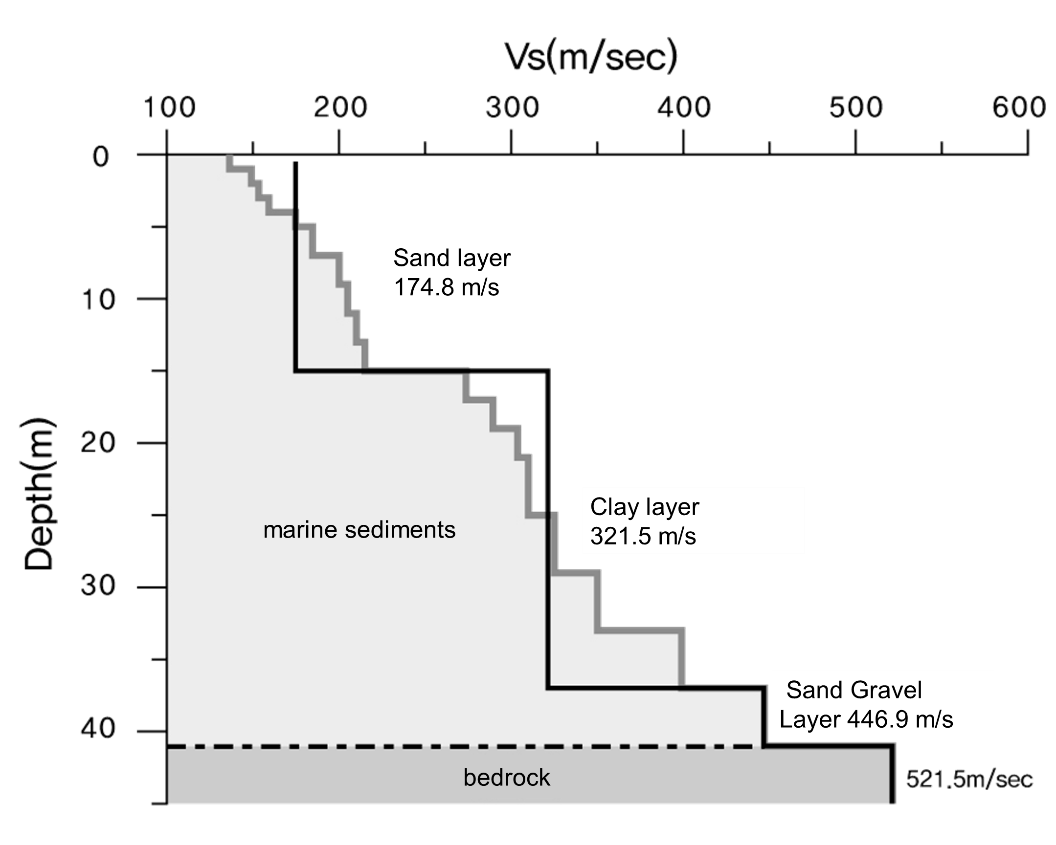


Supplemental Table 2: Time-averaged shear wave velocity (Vs) for profile

| Layer | | Depth (m) | description | Average Vs (m/sec) |
| --- | --- | --- | --- | --- |
| Marine sediment layer | Reclamation layer | 0 - 1.5 | Fine Sand |  |
|  | Sand layer | 1.5 – 15.0 | Sand | 174.8 |
|  | Clay layer | 15.0 - 37.0 | Silty clay, granule, shell | 321.5 |
| soft rock | Sand Gravel layer | 37.0 - 41.0 | Mudstone (cracks and joints, fracture zone) | 446.9 |
| bedrock | Hard rock | 41.0 - 48.0 | Mudstone | 521.5 |
